# Development of Light Powered Sensor Networks for Thermal Comfort Measurement

**DOI:** 10.3390/s8106417

**Published:** 2008-10-16

**Authors:** Dasheng Lee

**Affiliations:** Department of Energy and Refrigerating Air-conditioning Engineering, National Taipei University of Technology, Taipei, Taiwan, 106; E-Mail: f11167@ntut.edu.tw; Tel.: +886-2-2771-2171; Fax: +886-2-2731-4919

**Keywords:** Thermal comfort, PMV, Light-powered sensor networks, Energy beacon enabled mode, CSV

## Abstract

Recent technological advances in wireless communications have enabled easy installation of sensor networks with air conditioning equipment control applications. However, the sensor node power supply, through either power lines or battery power, still presents obstacles to the distribution of the sensing systems. In this study, a novel sensor network, powered by the artificial light, was constructed to achieve wireless power transfer and wireless data communications for thermal comfort measurements. The sensing node integrates an IC-based temperature sensor, a radiation thermometer, a relative humidity sensor, a micro machined flow sensor and a microprocessor for predicting mean vote (PMV) calculation. The 935 MHz band RF module was employed for the wireless data communication with a specific protocol based on a special energy beacon enabled mode capable of achieving zero power consumption during the inactive periods of the nodes. A 5W spotlight, with a dual axis tilt platform, can power the distributed nodes over a distance of up to 5 meters. A special algorithm, the maximum entropy method, was developed to estimate the sensing quantity of climate parameters if the communication module did not receive any response from the distributed nodes within a certain time limit. The light-powered sensor networks were able to gather indoor comfort-sensing index levels in good agreement with the comfort-sensing vote (CSV) preferred by a human being and the experimental results within the environment suggested that the sensing system could be used in air conditioning systems to implement a comfort-optimal control strategy.

## 1. Introduction

The basic requirement of an air conditioning system is to provide a thermal comfort level agreeable to the people in the building. The seven-point psychophysical ASHRAE scale, predicted mean vote (PMV), was employed to determine the thermal comfort level [1, 2]. The comfort sensing indexes considered were metabolic rate, clothing, air velocity, air temperature, air temperature stratification, radiant temperature, relative humidity and turbulence intensity in the occupied zone. The PMV value lies within the - 3 to +3 range. Negative values stand for cold feelings while positive values for hot feelings. Zero value represents the condition where most occupants feel thermally comfortable. It is a well-recognized index for judging appropriate levels of comfort. Many advanced air-conditioning control methods were developed by incorporating PMV in their algorithms [3-8].

Better performances than existing controls, in terms of human thermal comfort and energy consumption, were expected with thermal comfort controls. Multi parameter data acquisition in thermal spaces has so far, been regarded as difficult to achieve, thus preventing provision of a controller able to work with the sensors to form a complete thermal comfort control system. This low-cost and wireless sensor technology offers an opportunity to realize a network of sensors for data acquisition in space [9]. Recently, an open networking technology was reported to handle real-time PMV measurements [10]. The attributes that were offered by the system, such as low cost, small dimensions, ease of installation, easy and low-cost operation, ease of retrofit in existing spaces and independence from particular manufacturers were announced in these studies. However, sensing nodes still need electric power supplies to operate and the need for power line distribution or built-in battery power conflicts with the appeal of easy installation and retrofit in spaces.

In this study, a sensing system powered by artificial light was proposed to construct a wireless sensor network for thermal comfort measurement. The system consists of a spotlight, a communication module and a sensor node equipped with photovoltaic cells, a microprocessor with thermal sensors including a micro machined flow sensor designed and fabricated by our lab for accurate PMV calculation. The communication module, synchronized with a controllable spotlight, not only collects the sensing nodes data but also can power the distributed nodes through the light ray. This novel concept was expected to provide wireless data communication and wireless power transmission to the sensor node. Since the node does not need power lines or battery power supply, simple sensor node distribution for thermal comfort measurements could be implemented. The following sections present the system's architecture, hardware and software organization.

## 2. Light powered sensor network architecture

**Figure 1.**
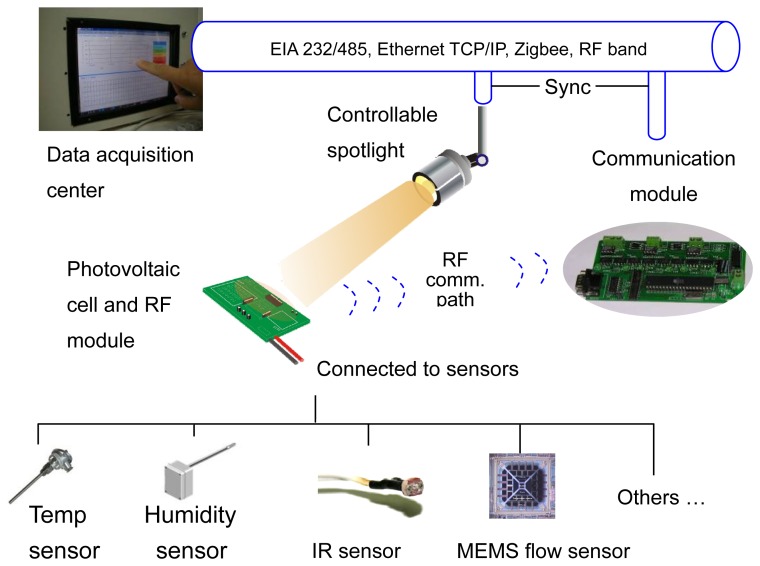
A schematic view of the light powered sensor networks.

A schematic view of the whole system is shown in Figure 1. The spotlight mounted on the dual axis tilt platform can project light onto the remote sensor networks node. The sensor nodes, comprised of the sensor, a photovoltaic cell and radio frequency (RF) devices, were distributed in space to collect the measured indoor environment parameters. The photovoltaic cell was able to convert the optical energy from the spotlight into electric current for powering the sensor and the circuits on the node. There was no power source on the node module. The light powered sensor network node delivers data through a specific RF communication protocol. With an ARM code microcontroller chip and the 8-channel low voltage differential signaling device, an embedded system was constructed to perform the sensor signal processing and the self-defined task of data communication services. Using a homemade communication module, the PC based data acquisition center was able to collect data from sensor nodes through RF communication. The other interfaces, including EIA RS 232/485 serial communication, TCP/IP protocol, Zigbee [11], are also integrated in the module for communications to the other equipment such as the power meter. The 935 MHz band was chosen for RF communication and a special energy beacon enabled mode was proposed to realize the special power architecture of the light powered sensor networks.

Traditional wireless sensor networks were operated in beacon enabled mode for communication synchronization. When a sensor node in the network is woken up and has a packet to transmit, it activates its receiver in order to synchronize with the beacon. If the node’s buffer is empty, it will return to sleep mode. The sensor node needs a continuous power supply to await the beacon’s trigger signal from the remote to indicate activation [12]. For the light powered sensor networks, the energy beacon enabled mode was proposed. Controlled synchronization between the spotlight projection and data communication ensured the energy beacon was able to send its transmission to the distributed sensor nodes. The spotlight projects a light beam onto the distributed sensor networks nodes and the photovoltaic current wakes up the sleeping node. This energy delivery acts as the communication beacon and not only synchronizes the data transfer but also provides the sensor node’s working power. Since only one node is woken up by the spotlight projection, no special collision prevention algorithm is necessary for communication and the simple packet format yields low consumption power for data communication. The working power for the sensor node relies only on the energy provided by the single spotlight projection. Moreover, the sensor node does not consume any power while sleeping.

### 2.1. Sensor node

**Figure 2.**
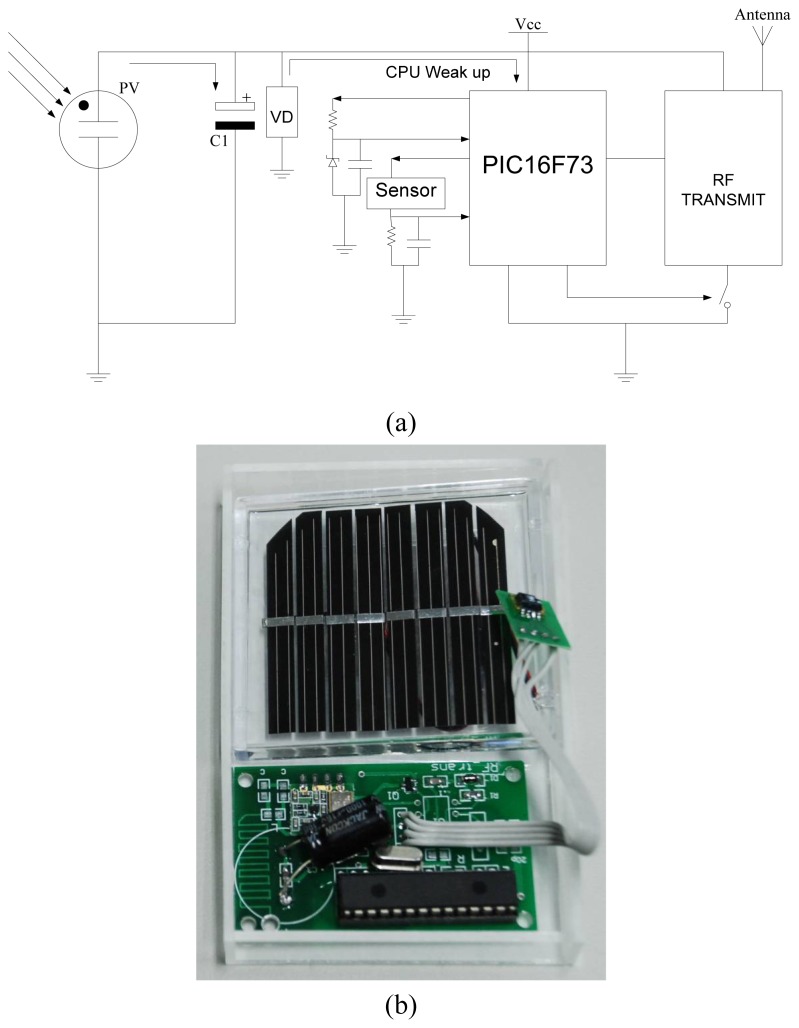
The circuit design of the light powered sensor networks node (a) and (b) a photograph.

Figure 2(a) shows the circuit design of the sensor node and 2(b) shows a photograph of the prototype. A 60 mm × 65 mm single crystal photovoltaic cell module panel was mounted on a board to transfer optical energy into the sensor nodes driving power. The solar cells were approximately 10-12% efficient, which was selected to allow the module to function at the light levels provided by the spotlight. [13] With respect to the different driving voltages, the current supply from the photovoltaic cell was estimated by:

(1) $$I=I_{\text{sc}}‐(20.91\cdot I_{\text{sc}}+5.37\times 10^{-3})\cdot {\left(V/V_{\text{oc}}\right)}^{8}$$

where the symbol, I, denotes the current, V is the sensor nodes driving voltage; I_sc_ and V_oc_ are the short circuit currents and the open circuit voltage of the photovoltaic cell, respectively. The maximum short circuit current supply was 150 mA and the open circuit voltage was up to 2.5 V. The light powered sensor networks relied on light exposure to supply working power. Based on Eq. 1, the minimum light intensity of the spotlight for driving the sensor node was determined and the power supply, with respect to different light reception intensities, was estimated.

To get the values of a PMV index in an indoor environment, it was necessary to know the temperature, relative humidity and airflow. Therefore, an IC-based temperature sensor, a relative humidity sensor, a radiation thermometer and a micro-machined flow sensor were integrated on the sensor nodes module. The LM335 temperature sensor [14] was employed for temperature sensing over a temperature range of −55°C to +150°C. The IC-based sensor had a breakdown voltage directly proportional to its absolute temperature at +10 mV/°K. When calibrated at 25°C the LM135 had typically less than 1°C error over a 100°C measurement range. This device consumed 400 μA of electric power at 2.5 V. The relative humidity sensing is performed by the Sensirion SHT1x [15]. It read out humidity values through a two-wire digital serial interface. Its relative humidity sensing accuracy was +/- 3.5% in a range of 20% to 80%. The power consumption was 550 μA at 2.5V. The radiative temperature was estimated by the readings of the infrared sensor [16]. Its accuracy level was +/- 2% of the reading range, which yielded 1.5 to 4.5 °C uncertainty. Power consumption of the infrared sensor and the signal processing circuits was less than 1 mA at 2.5V. A microprocessor, PIC16F73, processed the sensor input data and translated it into the packet-based message for wireless transmission. The RF transmission module, coupled to a chip antenna, transmitted the data through 935 MHz ISM band after receiving the energy beacon from the spotlight. The sensor node consumed zero power in standby stage and the communication operation required 90 mW of continuous power supply.

The thermal comfort was affected by the environmental air speed. The ISO regulation 7730 [2] recommends PMV values of between +0.5 and -0.5 to maintain thermal comfort with an advised air speed of less than 0.3 m/sec. In order to detect low speed airflow, an integrated flow sensor with microstructures and a mixed signal processor was developed.

### 2.2. MEMS flow sensor

**Figure 3.**
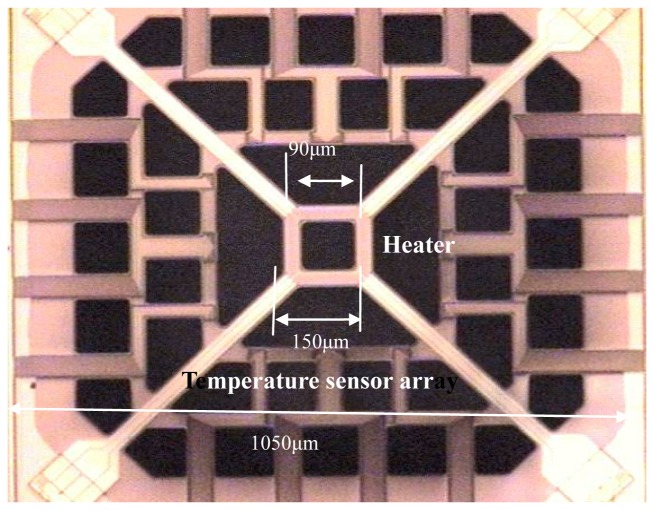

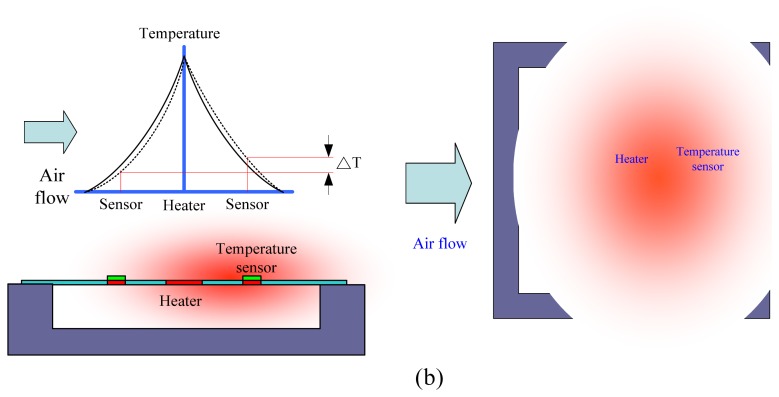
An enlarged photograph of microstructure of the MEMS flow sensor (a) and its working principals for low speed airflow measurement (b).

The microstructure of the flow sensor is shown in Figure 3(a). A square heater source was located at the center of a square polysilicon thin film with a cavity underneath. The square heater was made of polysilicon, with the dimensions as shown in Figure 3(a), and the gas in the cavity provided good thermal insulation when the electric power was supplied to the heater. Equally spaced aluminum-polysilicon thermopiles surrounding the heater were located at opposite sides, in both the x-and y-directions of the heater. These thermopiles served as array sensors detecting the temperature differences between the opposite sides of the heater. The outer zone of the die was the mixed signal processor. The whole chip, which was packaged on the backside and lateral sides, had a size of 5 mm × 5 mm × 2 mm.

Figure 3(b) illustrates the operating principle of the MEMS flow sensor. Once electric power is supplied to the heater, a thermal plume is generated in the air atmosphere, as shown. As the chip was positioned against the flow, the thermal plume was pushed towards the temperature sensors array and the temperature difference was converted into voltage to determine the airflow rate. The temperature sensors arrayed on four sides, gave the output voltages in both the x-axis and y-axis and the velocity components parallel to the chip plant were measured. The MEMS flow sensor measures low airflow speeds ranging between 0.1 m/s and 0.45 m/s. The sensitivity of the flow sensor was indicated as 0.7 mV/ m/s and 0.001 m/s velocity measurement resolution was achieved.

The sensor was utilized to monitor the airflow condition in the thermal environment. Generating the thermal plume and analyzing the airflow speed required 2 mA at 2.5 V power supply. Although a higher power consumption than the other sensors, the MEMS flow sensor provided the solution, which met the requirement of small dimensions for the distributed sensor node and the high-resolution airflow speed analysis for accurate PMV measurement.

### 2.3. Focusing spotlight

**Figure 4.**
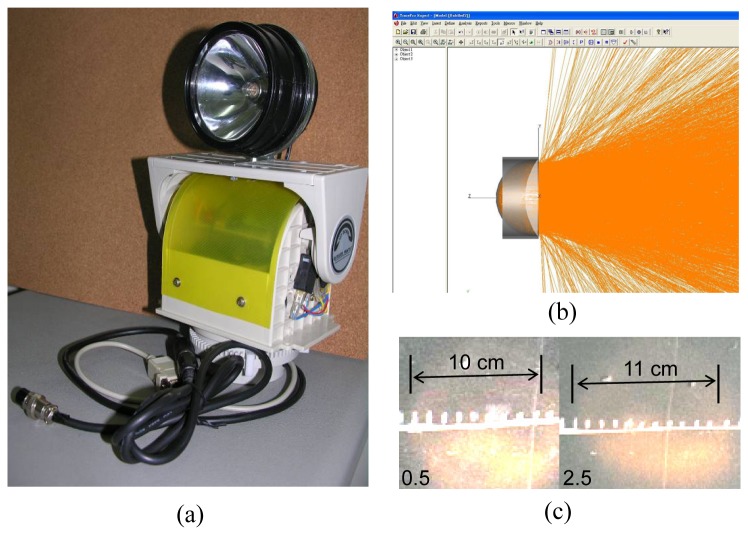
The photograph of the focusing spotlight (a) and the simulation results of the spotlight focusing design (b). The projection patterns of 0.5 meter distance and 2.5 meters away are shown in (c).

The focusing spotlight, shown in Figure 4(a), was employed to project optical energy onto the remote sensors network node. The required projection distance was set to 2.5 meters, which can cover the range for a practical air conditioning area. Two sets or more of spotlights were employed to extend the measurement distance for a large thermal environment. The intensity of the light should be kept as high as possible to ensure that the exposure can provide enough energy to support the sensor nodes processes of sequence sensing, i.e. the sensor with the highest power consumption can be switched on from a single light projection. According to the sensing components power requirements, described above, the minimum light intensity requirement for driving the sensor node was calculated by Eq. 1. We used TracePro® to simulate the light ray path and estimate the projections intensity with respect to the distance for the spotlight design. The results are shown in Figure 4(b). An elliptic shape reflector and a collimate lens were employed to retain the light divergence angle of less than 30 degrees. The small, solid angle of divergence yielded low light intensity decreases over the long-distance; thereby ensuring optical energy was transferred to the sensor node.

Using a 5W bulb, the required projection distance can be achieved with a retainable light intensity of 1,500 lux. The projection patterns of 0.5 and 2.5 meters distance are shown in Figure 4(c), respectively. Due to the small divergence angle, the actual projection distance of the spotlight was farther than expected. The long projection distance makes the system applicable to collect thermal comfort information for large spaces.

Although the spotlight can achieve a high intensity light projection, the actual optical power transferred to the remote sensor node was influenced by the variables of the transmission path, therefore shielding may have occurred randomly. In order to enable the sensor node to work, the photonic energy was not directly used to drive the circuit but stored in a capacitor for regulating the power supply. However, the shielding caused by the occupants' motion may interrupt the energy transfer to the sensor node and the data will be lost. Comparing with traditional wireless sensor networks, the data lost from the light powered sensor networks may be caused by not only the RF wave interruption but also the light beam shielding. How to solve the data loss problem was the issue for the application of the light powered sensor networks.

### 2.4. Maximum entropy method

Wireless data communication presents the problem of data loss if the RF wave propagation is interfered with. To ensure the communication module receives the whole information packet sent by the distribution nodes, a specific protocol packet is encoded to attach to the “End of File” stream at each communication event. If the communication module does not receive the ending stream, it will request the distribution node to resend to complete one successful data communication.

Wireless power transmission has a similar problem of data loss, but its issue is energy loss. Due to the occupants' actions, the projection light may be randomly shielded. If the system is on hold for every sensor node feedback, the long lag will dominate the communication and that yields data transmissions in an inefficient way.

A special algorithm, the maximum entropy method, was developed to estimate the sensing quantity of climate parameters if the communication module did not receive any response from the distribution node within a certain time limit. Data extrapolation was also employed in this study to give the estimated values by the polynomial fitting. Although it was a simple way, such estimation does not consider the system characters and it was highly influenced by sensor noise. The estimated results obtained from the proposed algorithm were compared with those from the data extrapolation to illustrate the advantages of the maximum entropy method.

The maximum entropy method relied on the assumption that the present time segment data could be determined according to the previous measurements, plus a deviation caused by white noise. This is expressed by [17]:

(2) $$X_{n}=-\sum_{m=1}^{M}a_{m}X_{n-m}+E_{n}$$

where E_n_ denotes the random deviation with a flat spectrum like the white noise and X_n-m_ denotes the previous measured data. With the weighting factor, a_m_, the present measurement, X_n_, could be estimated. M is the data points for estimation.

By rearranging Eq. 2 and times all terms by Xn, we got:

(3) $$X_{n}\cdot X_{n}+a_{1}X_{n-1}\cdot X_{n}+a_{2}X_{n-2}\cdot X_{n}+\cdots +a_{M}X_{n-M}\cdot X_{n}=E\cdot X_{n}$$

Eq. 3 is expanded by shifting n in time and integrating. The expanded polynomials can be expressed by:

(4) $$C_{\text{XX}}(0)+a_{1}C_{\text{XX}}(1)+a_{2}C_{\text{XX}}(2)+\cdots +a_{M}C_{\text{XX}}(M)=P$$

Cxx is the discrete auto correlation of X over time shift from zero to M. P is the integral of the estimation error, times the measured values.

Then we multiplied Eq. 2 by X_n-k_ and expand the time shift, the results are illustrated by:

(5) $$C_{\text{XX}}(k)+a_{1}C_{\text{XX}}(k-1)+a_{2}C_{\text{XX}}(k-2)+\cdots +a_{M}C_{\text{XX}}(k-M)=0$$

Different from Eq. 4, this equation equals to zero. This was due to the error being in random and the correlation to other time segments should be zero.

Combining Eq. 4 and Eq. 5, the following matrix was derived:

(6) $$\left[\begin{array}{cccc} C_{\text{XX}}(0) & C_{\text{XX}}(1) & \cdots & C_{\text{XX}}(M)\\ C_{\text{XX}}(1) & C_{\text{XX}}(0) & \cdots & C_{\text{XX}}(M-1)\\ \vdots & \vdots & \vdots \\ C_{\text{XX}}(M) & C_{\text{XX}}(M-1) & \cdots & C_{\text{XX}}(0) \end{array}\right]\;\left[\begin{array}{c} 1\\ a_{1}\\ \vdots \\ a_{M} \end{array}\right]=\left[\begin{array}{c} P\\ 0\\ \vdots \\ 0 \end{array}\right]$$

In Eq.6, we calculated the weighting coefficients with respect to each time segment. If the data loss was in one segment, the climate parameters could be estimated by the forward prediction, as follows;

(7) $$\text{fp}=\sum_{i=0}^{M}a_{i}X_{n-i}(M+1\leq \;n\;\leq N,a_{0}=1)$$

The forward predicted value, fp, was used as the output of the sensing temperature, radiative temperature, humidity and wind speed as no response could be obtained from the distributed sensor nodes. Since the estimation gave additional data points during no input conditions, information entropy was increased and that is why the method was called maximum entropy. Based on the static model, the system characters were identified. During data recording periods, the model was adjusted continuously with respect to the estimation error. Since auto correlation was introduced into the calculation, the noise was rejected and precise data estimations could be expected.

### 2.5. PMV measurement

According to the feedback from the distributed sensor nodes and the estimated data, PMV values could be calculated by [18]:

(8) $$\text{PMV}=\left(0.303e^{-0.36M}+0.028\right)\left\{\begin{array}{l} \left(M-W\right)\\ -3.05\times 10^{-3}[5733-6.99(M-W)-P_{a}]\\ -0.42[(M-W)-58.15]\\ -1.7\times 10^{-5}M(5867-P_{a})\\ -0.0014M(34-t_{a})\\ -3.96\times 10^{-8}\;f_{\text{cl}}\left[{\left(t_{\text{cl}}+273\right)}^{4}-{\left(t_{r}+273\right)}^{4}\right]\\ -f_{\text{cl}}h_{c}\;(t_{\text{cl}}-t_{a}) \end{array}\right\}$$

Where:

tcl=35.7−0.028(M−W)−Icl{3.96×10−8fcl[(tcl+273)4−(tr+273)4)]+fclhc(tcl−ta)}hc=〈2.38(tcl−ta)0.25for2.38(tcl−ta)0.25≥12.1Va12.1Vafor2.38(tcl−ta)0.25≤12.1Vafcl=〈1.00+1.29IclforIcl≥0.078m2C°/W1.05+0.645IclforIcl≤0.078m2C°/W

M indicated the metabolic rate and W was the external work. The parameter, f_cl_, is the ratio of a man's surface area while clothed to his surface area while nude. That value was calculated by the thermal resistance of a man's clothing coefficient, I_cl_. These values could be estimated according the occupants' conditions in the thermal environment. The ta was the air temperature measured, tr was the mean radiant temperature, V_a_ denoted the air velocity and P_a_ was the partial water vapor pressure. These measured parameters were provided by the distributed sensor node. The convective heat transfer coefficient, h_c_, was determined with respect to the air flow conditions in space. By considering the measured parameters and the occupants’ conditions, the surface temperature of clothing, t_cl_, was calculated and the predicted comfort index, PMV, could be determined.

In order to investigate the characteristics of these sensors in response to temperature, humidity, and airflow, among other things, PMV measurements were carried out in a moderated space. The PMV values were calculated according to the feedback of the light powered sensor networks and the measurements by the commercial instrument, respectively. A Testo 400 multifunction instrument was employed to measure the thermal comfort parameters for the controlled experiment. The probes had been calibrated for adjustment capabilities of temperature measurement with an accuracy to <0.1 °C, relative humidity up to ± 1.0 % and the velocity measurements in ± 1.0 % range of 0 to 20 m/s. By comparing the measured data from the light powered sensor networks and the multifunction instrument, the accuracies of the fabricated sensors equipped on the sensor node were examined.

Actual thermal comfort measurements using the light powered sensor networks were performed in a computer classroom. Thirty-eight participants were exposed in this thermal environment. Then input data regarding sex, height, weight, and clothing information was entered into the data acquisition center, which then voted thermal comfort. At the same time, the light powered sensor networks collected comfort relative parameters and calculated the PMV. The cross correlations of the comfort sensing vote (CSV) and the PMV values were investigated to verify the effectiveness of the data processing algorithm and working principals of the light powered sensor networks.

## 3. Results and Discussion

**Figure 5.**
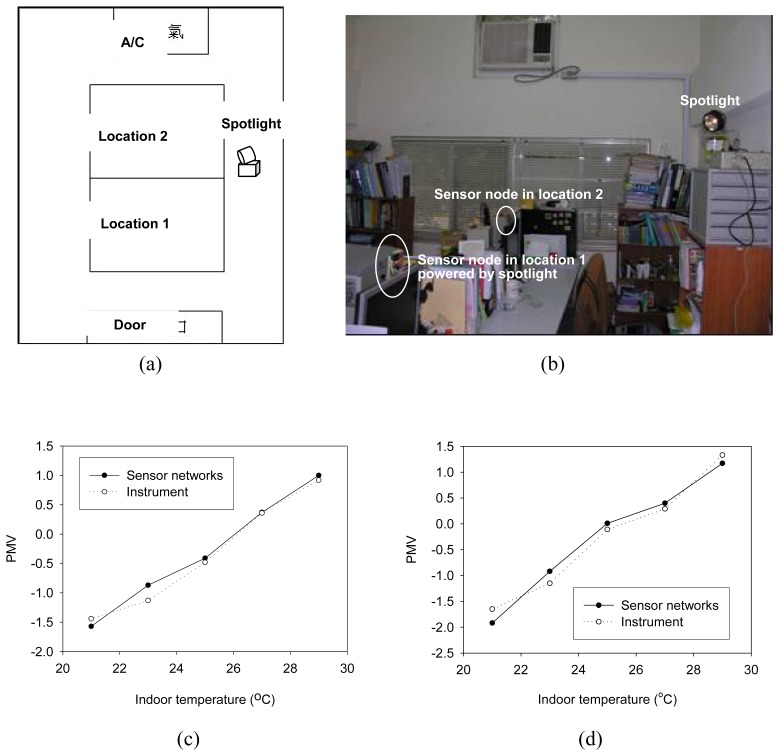
PMV indices in the simulated thermal environment. The schematic view of the space arrangements is shown in (a). The photograph of the light powered sensor networks assembled in the thermal environment is shown in (b). Panel (c) and (d) illustrate the PMV indices obtained by the commercial instrument and the light powered sensor networks at the location 1 and 2 respectively.

In a 3.2 meters wide and 5.25 meters long laboratory, we increased the air conditioner temperature setting from 21 °C to 29 °C and measured PMV by both light powered sensor networks and the commercial instrument. Figure 5(a) shows the detection locations with respect to the rooms furnishings schematically and Figure 5(b) shows the photograph of the sensor networks distribution in space. Since the interested area was limited in a 5 meter square area, one spotlight was used to power the distribution sensor node. Detecting location 1 was on the central of table, far from the air conditioner. Location 2 was closer to the air conditioner and the sensor node was just behind the fan outlet, as shown in Figure 5(b). The PMV measurements at these two locations provided the airflow speeds influence upon thermal comfort. Since it was a controlled experiment for comparing instrument performances, no people participated in this test. The spotlight control, synchronized with the communication module, enabled the sensor nodes to collect environmental information and a zero data loss rate was achieved. Figures 5(c) and (d) illustrate the PMV indices obtained by the commercial instrument and the light powered sensor networks at locations 1 and 2, respectively. As the low temperature setting was down to 21 °C, PMV values measured at location 2 were around ‐2. At location 1, PMV values were around ‐1.5 and this gave a slight cooling feeling. Both instruments readouts corresponded to the cooling feeling required for a human’s comfort.

PMV values obtained from the light powered sensor networks complied with those obtained by the commercial instrument. Less than 5% deviation was observed. This illustrated that temperature sensing and humidity detecting performed by the sensor nodes had similar accuracy as those obtained in the experimental facility. Moreover, the influence on thermal comfort was identified by the MEMS flow sensor. The microstructure flow sensor designed and fabricated by our lab gave reliable measurements of the airflow rate in the range of heating, ventilation, and air conditioning (HVAC) equipment controls.

**Figure 6.**
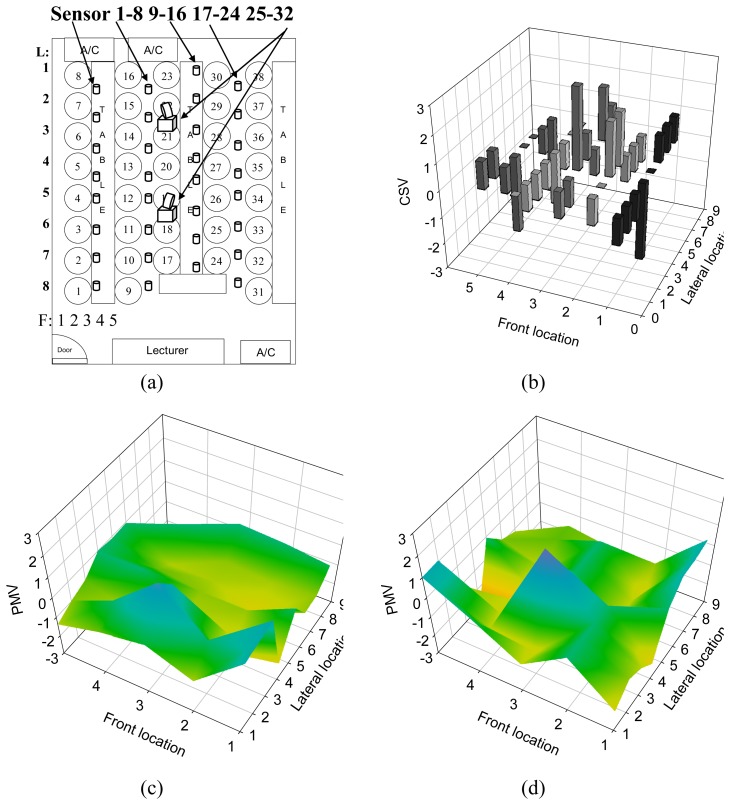
The comparison of CSV obtained by human vote and PMV measured in a practical space. Panel (a) shows the arrangements of the space, a computer classroom with thirty eight seats for participants. Panel (b) gives the vote of participants. The PMV measured results in space that obtained by the sensor networks with the extrapolated estimation and the maximum entropy method are shown in (c) and (d) respectively.

Comfort sensing vote was carried out in a 7.5 meter wide by 8.35 meter long computer classroom containing thirty eight people. Figure 6(a) shows the participants' locations and sensor nodes distributed condition schematically. Front location and the lateral location can be referred to the marker shown on the figure. Participants' vote results are shown in Figure 6(b) and the comfort rating was evaluated in the range of ‐3 to +3 with integral increments from cold, cool, slightly cool, neutral, slightly warm, warm, and hot. At the same time, PMV measurements were performed to predict thermal comfort levels by the thirty two sensor nodes distributed in space. Two spotlight sets were employed to enable the distributed nodes for PMV measurements in the testing area. However, the actual projection distance of the spotlight was farther than the expected and one spotlight was able to power the sensor node from further away than 5 meters in the test. During the experiment, participants were encouraged to sit or walk through the passenger way, which meant the projected light beam from the spotlight was occasionally shielded. The data loss due to the failed energy transmission happened in random. The overall data loss rate was approximately 15%. The comfort sensing system had to estimate the measured parameters and fill the data points for completing PMV calculation.

Two different algorithms, extrapolation and maximum entropy methods, were employed to estimate the data loss. Figure 6(c) and (d) show the PMV indices distribution in space that were obtained by the sensor networks with the extrapolated estimation and the maximum entropy method, respectively. It is clear from Figure 6 (b) and (d) that the output values from the light powered sensor networks were in good agreement with the CSV of a human being. The cross correlation of PMV and CSV was 0.95. On the other hand, with the simple extrapolation method, the PMV measurements, by the sensor networks, gave poor results and the cross correlation was down to 0.78. Although it is still good enough for comfort vote prediction, the maximum entropy method was proposed to give more accurate predictions for human comfort sensing.

In the case of the computer classroom, the participants sitting near the air conditioner located in the front of the classroom felt cold, but those behind them felt hot. The PMV values for the longitude direction of the front location 5 indicated neutral thermal comfort. The airflow measurements therefore gave advisable air circulation conditions for that location. The cold air flow from the air conditioner spread out in front of the seats providing no direct thermal impact on the occupants. Only two participants felt slight warmth since they remained in the room’s corner. Another two air conditioners, located at the back of the room gave different cold air distribution than the others in the room. Most of the people who stayed in this air conditioning area felt cold, due to the cold air impacts. However, the last three occupants near the door felt warm. Obviously, this was caused by the drag effects of the occupants sitting in front of them. With the exception of the single, previous result, the feedback from the sensor networks reported high relative humidity at that location and that may have been due to the outlet air leakage through the door. In the center of the room, occupants were uncomfortable and even hot, thermal fluctuations were indicated there. This may have been caused by an impinging flow effect, since the air conditioners were arranged face to face. Using the light powered sensor networks, the details of the thermal environment were investigated and the causes of all uncomfortable feelings were identified. Experimental results clearly suggest this sensing system can be applied in air conditioning systems as a practical means of monitoring PMV and CSV indices.

## 4. Conclusions

Combining optical design, MEMS technologies, and wireless communication, light powered sensor networks were constructed to perform environmental thermal comfort measurements. This novel comfort sensing system can achieve wireless data communication and wireless power transmission for distributed sensing. Although retaining the advantage of easy installation, the probability of data loss was increased due to the power transfer method, through light, may occasionally be impeded by the occupants in the space. The estimation algorithm by the maximum entropy method was developed to solve the data loss problem. Thirty eight people have conducted PMV measurements with light powered networks of the degree of comfort in a 7.5 meter wide by 8.35 meter long computer classroom. The cross correlation between the result of PMV by light powered wireless networks and comfort sensing vote by people is up to 0.95. The results illustrate the estimation output of the maximum entropy method gave high accurate predictions for human comfort sensing. The study and development of new type of sensor networks with wireless communication and wireless power transfer through light, though the efficiency is not necessarily good but the principal effectively works, is proposed to construct air conditioning control system based on the comfort degree data. By connecting the sensor networks to the HVAC equipment actuators, energy-consumption control could be realized through the feedback of the thermal comfort information in the space. The light powered sensor networks described in this study are being installed in the chain stores. The energy saving control started in August of 2008. More experimental data was collected and will be reported in the near future.
